# Study protocol: associations between hormonal profile and physical and cognitive functions in middle-aged men—a one-year cohort follow-up study

**DOI:** 10.3389/fpubh.2025.1654077

**Published:** 2025-09-01

**Authors:** Dan Keren, Abigail Goshen, Tzipi Strauss, Shmuel Springer

**Affiliations:** ^1^The Neuromuscular and Human Performance Laboratory, Ariel University, Department of Physical Therapy, Faculty of Health Sciences, Ariel, Israel; ^2^Sheba Longevity Center, Sheba Medical Center, Tel HaShomer, Ramat Gan, Israel; ^3^Gray Faculty of Medical and Health Sciences, Tel Aviv University, Tel Aviv, Israel

**Keywords:** midlife, males, sex-hormones, testosterone, intrinsic-capacity, cognitive, physical, function

## Abstract

**Background:**

Middle age (45–64 years) is a critical yet under-researched phase of aging, during which the first signs of physical, cognitive, and hormonal decline often emerge. In men, testosterone (T) levels begin to decrease starting in the fourth decade of life. However, the relationship between T levels and functional abilities in midlife men remains poorly understood. A better understanding of how physical, cognitive, and hormonal domains interact during this transitional phase is essential for developing targeted interventions to promote healthy aging. Using advanced assessment tools, we propose a study aimed to clarify this association in middle-aged men.

**Methods:**

This is a one-year follow-up cohort study. Two hundred healthy middle-aged men will undergo comprehensive assessments, including blood tests, body composition analysis, balance and strength testing, and computerized cognitive evaluation. Half of the sample will perform a subset of the balance and strength tests with a smartphone on their back to obtain accelerometry based measures that have been shown to detect subtle functional changes in midlife. T levels will be measured again after 6 months, and a full assessment will be reperformed after 1 year.

**Expected results:**

We anticipate that men with higher T levels will have better physical and cognitive function, with these associations persisting over a one-year period.

**Conclusion:**

This study addresses a critical research gap by clarifying the relationships between hormonal, physical, and cognitive function during midlife. If significant associations are identified, T level assessments could be integrated into routine preclinical screenings alongside physical and cognitive evaluations.

## 1 Introduction

Middle age, also known as midlife, is usually defined as the phase of life between the ages of 45 and 64. This period plays a central role in life and is described as the key phase between adulthood and the later stages of life (1). In midlife, human performance can vary greatly from person to person, as physical and cognitive functions begin to decline (2). Up to 20% of the middle-aged population report mobility difficulties, despite having only mild health problems (3). While cognitive abilities such as accumulated knowledge and vocabulary, are often preserved in midlife, complex problem-solving, processing speed and memory may decline (1). These age-related changes can affect independence and quality of life, and increase the risk for unhealthy aging. However, most research on the effects of aging on physical and cognitive function has focused on the older population (4, 5). A better and deeper understanding of the early stages of aging could help to identify individuals who are at higher risk of accelerated decline and allow for the implementation of early interventions to promote healthier aging.

To better understand the mechanisms of aging, it is important to consider the different age-related trajectories of men and women. Women tend to live longer but are more prone to frailty and disability, while men are more likely to be affected by cardiovascular disease and cognitive decline despite maintaining better physical function in midlife (6). These sex-specific differences, which are shaped by biological and hormonal factors, underscore the importance of sex-specific screening tools. Furthermore, while midlife women experience a well-defined hormonal transition with menopause whose health effects have been widely studied, hormonal changes in men, particularly the gradual decline in testosterone (T), are more variable, the presence of associated symptoms does not always correspond to T levels, and have been far less researched (7). Studies indicate that approximately 40% of men experience clinically significant T decline in midlife (8). However, the definition of this period, often referred to as ‘andropause’, remains controversial (9), possibly due to the limited understanding of hormonal changes in men and their relationship to functional abilities.

Low T levels are associated with increased fat mass, decreased muscle mass and metabolic disorders, which can start a feedback loop that further suppresses T levels (10). However, the relationship with physical performance is inconsistent (11). Most studies that have examined this relationship have focused on older men and assessed function using tests such as the sit-to-stand test (STS), tandem stance and walking speed, for which there are no established normative values for middle age. Furthermore, these tests are often not challenging enough and have ceiling effects, so subtle changes in this population may not be detected (12). Recent studies using advanced and more sensitive performance testing tools such as smartphone-based accelerometry in the STS, TUG and Single Leg Stance (SLS) tests have shown that subtle preclinical functional changes can be detected as early as midlife (13, 14). Nevertheless, further research is needed to test these tools in larger samples and in a longitudinal study.

Studies examining the relationship between T levels and cognitive function in adult men have found that higher T levels are linked to a reduced risk of cognitive decline, dementia, and Alzheimer’s disease (15). Evidence suggests that T may protect brain function by slowing glucose metabolism decline and limiting beta-amyloid plaque accumulation associated with Alzheimer’s disease (15). Additionally, several studies have found a positive correlation between T levels and cognitive domains such as memory, attention, and executive function (11, 16). However, most of this research has focused on older adults, underscoring the need to investigate these associations in midlife to better understand the early stages of aging.

Beyond T, hormones such as sex hormone−binding globulin (SHBG), dehydroepiandrosterone sulfate (DHEA-S) and estradiol have also been studied for their role in age-related changes in physical and cognitive function. SHBG levels rise with age and are inversely associated with strength and cognitive function, though these associations often weaken after adjusting for confounders like age (11, 17, 18). DHEA-S correlates positively with executive function and memory in older men, but these associations have not been maintained in longitudinal models (19). DHEA-S is also positively associated with metrics like grip strength and gait speed, particularly in middle-aged men (20). Although there is evidence of an association between estradiol and body composition (21), this does not consistently translate to physical performance (21), and findings on association with cognitive function remain mixed (17, 22).

Modifiable lifestyle and psychological factors such as smoking, sleep quality, depression and anxiety, have also been associated with T levels, cognitive function, and physical performance (23–26). Addressing these factors in midlife research may help to better characterize the early signs of decline and support the development of timely, personalized interventions.

A deeper understanding of the interactions between physical, cognitive and hormonal domains during this transitional phase is essential for the development of personalized interventions to promote healthy aging. Therefore, the primary aims of this study are: (1) To examine associations between physical performance, cognitive function, T levels and self-reported androgen deficiency symptoms in middle-aged men and to assess the dynamics of these associations over 1 year. (2) To characterize physical and cognitive performance of middle-aged men and establish reference values for functional assessments. (3) To examine whether smartphone-based accelerometry correlates more strongly with T levels than other functional tests in middle-aged men. We hypothesize that men with higher T-levels will have better physical performance and cognitive function, that these associations will be maintained over a one-year follow-up period, and that the variables measured with smartphone-based accelerometry will show a stronger correlation with T-levels.

## 2 Methods

### 2.1 Study design and procedures

This is a one-year cohort follow-up study, which was approved by the Institutional Review Board of Sheba Medical Center (SMC-0761-23) and complies with the principles outlined in the Declaration of Helsinki.

**Figure 1:**
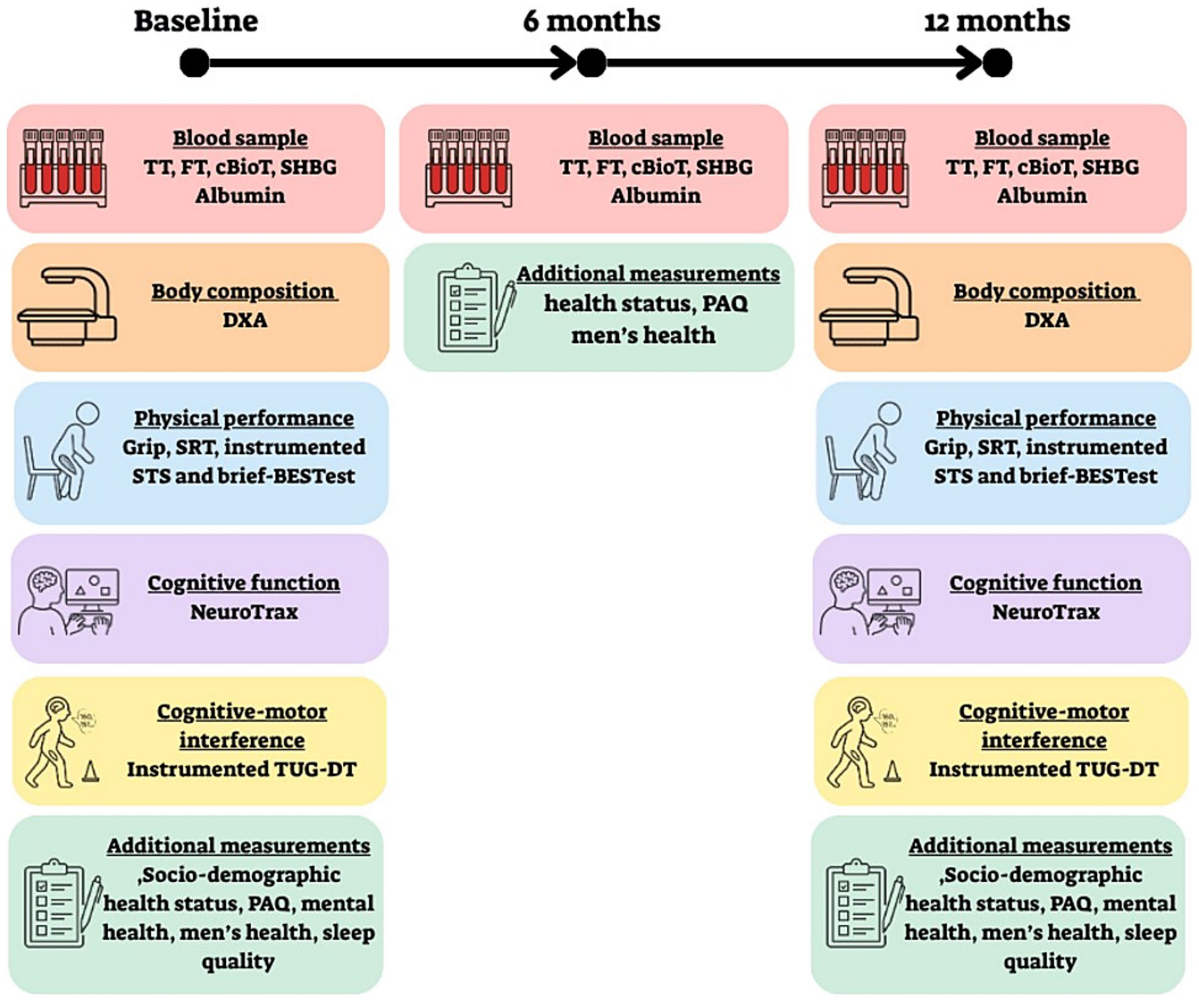
Description of the assessments to be performed at the three study time points (baseline, 6 months, and 12 months). TT, Total testosterone; FT, free testosterone; cBioT, Calculated bioavailable testosterone; SHBG, Sex hormone−binding globulin; DXA, Dual-energy X-ray Absorptiometry; SRT, Sitting-rising test; STS, Sit-to-stand; brief-BESTest, brief-balance evaluation systems test; NeuroTrax- computerized cognitive tests; TUG-DT, Timed up and go with dual task; PAQ, Physical activity Questionnaire.

Participants will be examined three times: at baseline, after 6 months and after 1 year. At baseline and at the one-year follow-up, they will undergo a comprehensive assessment including self-reported background questionnaires, blood sampling, physical and cognitive tests and body composition measurement. At the six-month follow-up, blood samples will be taken, and self-report questionnaires will be completed to monitor short-term hormonal and subjective health changes. A description of the assessments that will be performed at each of the three study time points is presented in Figure 1.

### 2.2 Participants

Recruitment will be conducted via center staff, social media, referrals from general practitioners or collaborators, and direct sign-up through the center’s website. Healthy community-dwelling men aged 50–65, able to walk outdoors without assistance, will be included. Participants presenting significant neurological, orthopaedic, cognitive, or visual impairments (e.g., age-related macular degeneration, glaucoma, diabetic retinopathy), psychiatric diagnoses or use of psychiatric medications, or chronic use of medications or supplements known to directly affect T levels (e.g., opioids, T supplements, androgen synthesis inhibitors, anabolic steroids), will be excluded.

The required sample size was calculated using G*Power software (version 3.1.9.4) for a multiple linear regression model with six predictors. Assuming a medium effect size (f^2^ = 0.15), an alpha level of 0.05, and a statistical power of 0.80, a minimum of 98 participants is required to detect a statistically significant association. To increase the reliability of the findings and to account for potential exclusions, data loss, and dropouts during the one-year follow-up, a total of 200 participants will be recruited. Physical performance, cognitive function and T levels will be assessed in all 200 participants. Half of the participants (n = 100) will perform a subset of the balance and strength tests with a smartphone worn on their back to obtain accelerometry based measures (i.e., instrumented physical assessments).

### 2.3 Data collection and outcome measures

#### 2.3.1 Hormone levels and symptoms

Albumin, SHBG, TT and FT levels will be measured from blood samples collected by a nurse in the early morning after a 12-h fast. The bioavailable T levels will be calculated (cBioT) from the known values of TT, SHBG, and albumin using a simple well-accepted formula (available from: http://www.issam.ch/freetesto.htm).

*Androgen deficiency symptoms* will be evaluated using the Androgen Deficiency in the Aging Male (ADAM) questionnaire and its quantitative version (qADAM). The ADAM and qADAM questionnaires include 10 binary and scaled items, respectively, and demonstrate high sensitivity (83.3–97%) but low specificity (19.7–60%) (27).

#### 2.3.2 Physical performance

All physical performance assessments will be conducted by experienced physical therapists.

*Grip strength* will be measured using the Exacta™ Hydraulic Hand Dynamometer (North Coast Medical, Inc., Morgan Hill, CA, USA). Participants will complete three maximal-effort trials per hand, alternating to prevent fatigue, with results averaged for each hand.

*The sitting-rising test (SRT)* will evaluate the ability to sit and rise from the floor with minimal support (score range: 0–10). Each use of support (e.g., hand or knee) deducts one point, and instability deducts half a point. Lower scores have been associated with increased all-cause mortality and reflect reduced musculoskeletal health (28).

*The 30-s STS test* will assess lower limb muscle strength. Participants will rise from a chair with wrists crossed over the chest and perform as many repetitions as possible within 30 s.

*Balance* will be assessed using the brief-balance evaluation systems test (brief-BESTest) (29). The test consists six sub-measures as follows: *Biomechanical Constraints* tested by hip/trunk lateral strength; *Stability Limits* tested by functional reach forward; *Anticipatory Postural Adjustments* tested by the ability to maintain single leg stance in each leg; *Reactive Postural Responses* tested by compensatory lateral stepping response in each side; *Sensory-Orientation* tested by stance on foam with eyes closed; and *Stability in Gait* tested by TUG. Two sub-measures (Anticipatory-Postural- Adjustments and Reactive-Postural-Responses) are scored bilaterally in each leg, resulting in eight test items. Each item scored between 0–3 and the maximal total score is 24 (i.e., a higher score indicates better balance performance).

*Cognitive-motor interface* will be assessed using the TUG dual task (TUG-DT), in which participants will perform the standard TUG test, while simultaneously counting backward in increments of three from a random three-digit number. The difference in total duration from the standard TUG will be calculated and used as an indicator of dual-task cost.

*Instrumented physical assessments* will be conducted in half of the sample (n = 100) during the STS, SLS, foam stance and TUG tests. A smartphone (Huawei Honor Play model; Android 10) will be attached to the participants’ lumbar spine near the body’s center of mass, and acceleration data will be recorded with the Phyphox application (RWTH Aachen University, Germany) using the same methodology as previously published (13, 14). For the STS, in addition to the number of repetitions measured in the standard test, the following parameters will be extracted using the smartphone: the average duration of rising phase, peak and average vertical velocity, the consistency of movement across repetitions using the dynamic time-warping (DTW) algorithm applied to the velocity profile, and the average lower limb muscle power during the sit to stand phase (14). Power (W) will be calculated using body mass (kg), vertical acceleration (aᵥ + 9.81 m/s^2^), and vertical velocity (vᵥ) during the rising phase, using the following formula:


Power[W]=Body mass[kg]·(av+9.81)[m/s2]·vv[m/s]

For the SLS and foam stance tests, the instrumented outcomes will include postural sway that will be quantified using the 95% confidence ellipse of the COM (COM-95%) trajectory (13). For the TUG, the turning phase sub-phase duration and the average angular velocity around the vertical axis during turning will be extracted (30). All the extracted data from the instrumented assessments will be processed using MATLAB (Mathworks, Inc.; version 9.12), and the vertical acceleration signal will be filtered with a 4th-order Butterworth low-pass filter with a cutoff frequency of 3 Hz.

#### 2.3.3 Cognitive function

Cognitive function will be assessed using the NeuroTrax™ computerized cognitive test battery (NeuroTrax™ Corp., Modiin, Israel), which has demonstrated high reliability and validity in diverse populations (31). The assessment will be administered under the supervision of a psychologist. The selected subtests target key domains relevant to aging: Verbal Memory (immediate and delayed recall of word pairs), Go/No-Go (attention and executive function via response inhibition and accuracy), Stroop (cognitive flexibility through interference control), and Problem-Solving Spatial (spatial reasoning and abstract thinking). Each test yields domain-specific scores and contributes to a global cognitive score, automatically calculated and standardized to an IQ-like scale (mean = 100, SD = 15), providing a comprehensive cognitive profile for each participant.

#### 2.3.4 Co-variants

**Figure 2:**
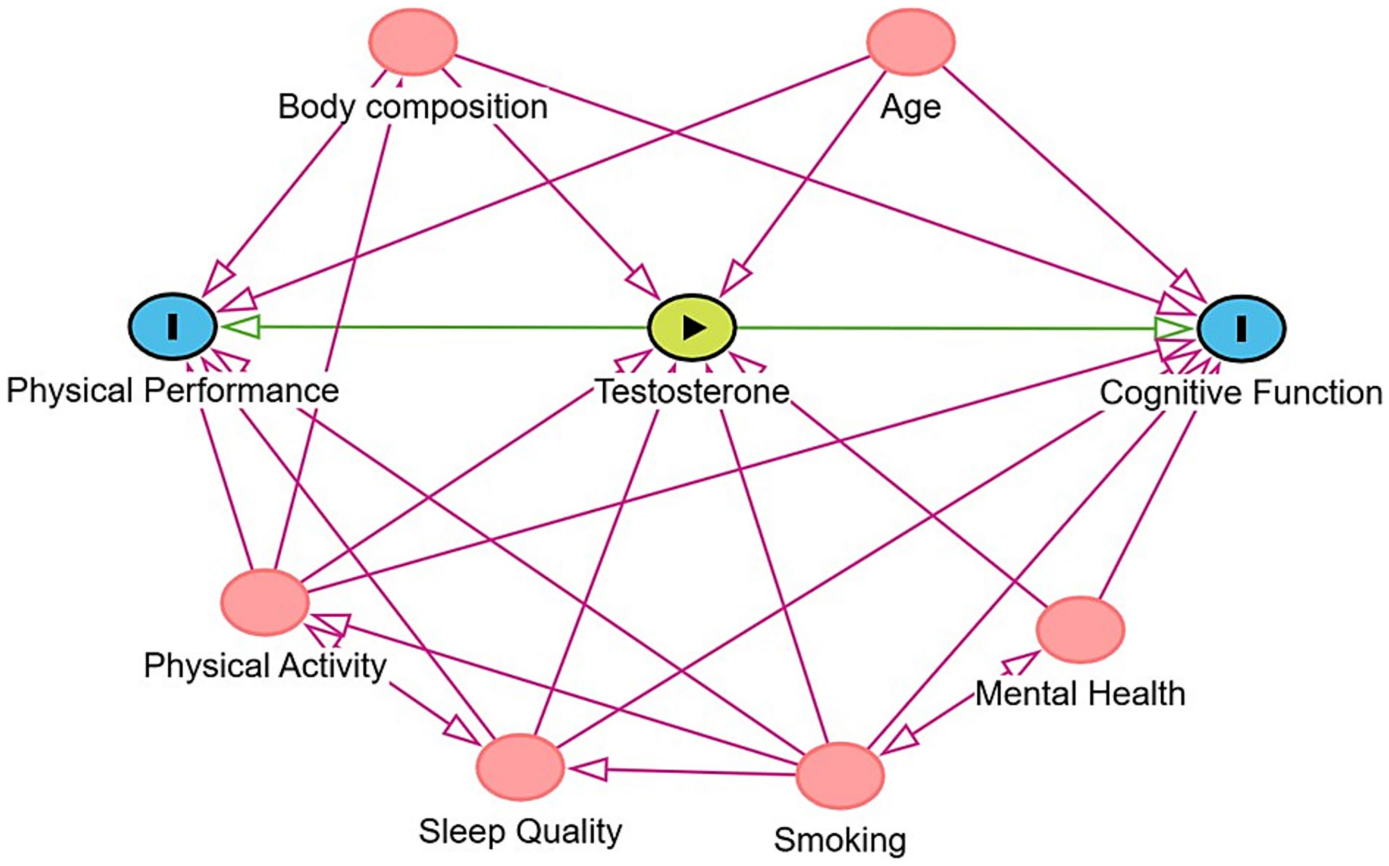
Directed Acyclic Graph (DAG) illustrating the hypothesized causal structure linking testosterone (green; exposure) to physical performance and cognitive function (blue; outcomes). The pink nodes represent variables identified in the literature as potentially affecting both exposure and outcomes, such as age, sleep quality and body composition. These variables were included for statistical adjustment to reduce confounding bias. The model was created using DAGitty.

To examine the association between T levels, physical performance and cognitive function, data on a set of relevant covariates will be collected. These include age, body composition, physical activity level, smoking status, sleep quality and mental health. The selection of these covariates was informed by a directed acyclic graph (DAG), constructed using DAGitty software (32). This approach helps to minimize bias by clarifying the underlying causal structure and identifying appropriate variables for adjustment. The resulting DAG guided the specification of the regression models by illustrating the potential pathways through which the variables may be interrelated, as shown in Figure 2.

*Participant backgrounds* will be assessed through a structured questionnaire covering sociodemographic and general health information. This will include items on income, education level, employment status, overall health, medication use, smoking status, alcohol consumption, family medical history, and self-reported health status and history.

*Physical activity* (PA) will be assessed using the widely used Baeck questionnaire, which covers work-related, sports, and non-sport leisure activities. Each section score is the average of frequency and intensity ratings on a five-point scale (33).

*Body composition* Weight and height will be measured to calculate BMI. Dual-energy X-ray absorptiometry (DXA; GE Healthcare; Madison, WI, USA) will be performed by a certified technician from the medical center to assess fat mass index (FMI), fat-free mass index (FFMI), and visceral adipose tissue (VAT).

*Sleep quality* and insomnia severity will be evaluated using the Insomnia Severity Index (ISI) (34). The ISI evaluated insomnia severity using a 5-point Likert scale for several questions (total scores 0–28), with higher scores indicating greater severity. The ISI demonstrates excellent reliability (Cronbach’s *α* = 0.90–0.91) and validates well compared to sleep diaries and psychological measures (34).

*Mental health* will be assessed using two widely recognized, reliable and validated tools: the Beck Anxiety Inventory (BAI) and the Beck Depression Inventory (BDI) (35, 36). Both include 21 items, each scored between zero to three. A total score of over 25 on the BAI or over 28 on the BDI indicates severe anxiety or depression (35, 36).

A summary of all the outcome measures in this study can be found in Table 1.

**Table 1:** Summary of outcome measures.

Domain	Assessment	Main outcome measures (units)
Physical performance	Grip strength	Average (kg)
	SRT	Total score
	STS	Number of repetitions (N)
	Smartphone instrumented STS (*n* = 100)	Power (W) Peak and average VV (rad/s) Movement consistency (DTW)
	brief-BESTest	Total score (0–24)
	Smartphone instrumented Brief-BESTest (*n* = 100)	COM-95% ellipse (m^2^)^2^ TUG phase durations (sec) Angular velocity (rad/s)
Cognitive-motor interface	TUG-DT	Dual-task cost (Δsec)
Cognitive function	NeuroTrax	Global cognitive score Verbal memory Attention Executive function
Hormones	Blood test	FT (pg/ml) & cBioT (ng/dL)
	Men’s Health Questionnaire	Binary outcome (positive/negative) Quantitative score (range: 10–50)
Additional	DXA	BMI, FMI (kg/m^2^)
	PAQ	Total score
	ISI questionnaire	Total score

SRT, sitting-rising test; STS, sit to stand; VV, vertical velocity; DTW, dynamic time-warping; BESTest, balance evaluation systems test; COM, center of mass; TUG, timed up and go; DT, dual task; FT, free testosterone; cBioT, calculated bioavailable testosterone; DXA, Dual-energy X-ray absorptiometry; FMI, fat mass index; VAT, visceral adipose tissue; ISI, Insomnia Severity Index; BAI, Beck anxiety inventory; BDI, Beck depression inventory.

### 2.4 Data analysis and statistics

The Shapiro–Wilk test will be employed to assess the normality of data distribution. Descriptive statistics will be presented as mean ± standard deviation (SD) or median with interquartile range (IQR) for continuous variables, and as frequencies and percentages for categorical variables. These analyses are intended to support the objective of establishing reference values for functional assessments in middle-aged men.

To address the first primary aim, Pearson or Spearman correlation coefficients will examine the relationship between physical performance, cognitive function and T levels (FT and cBioT) based on the baseline data. Variables showing moderate correlations (r ≥ 0.2) will be included in multiple linear regression models adjusting for the covariates. Additionally, within-subject changes in T levels, physical function, and cognitive performance over the one-year follow-up period will be assessed using paired t-tests or Wilcoxon signed-rank tests, depending on the distribution of the data. To further explore individual variability in these changes, linear regression models will be used to examine whether baseline characteristics (age, BMI, physical activity, sleep quality, and initial scores in each domain) are associated with the magnitude of change in outcomes. This analysis will help to identify baseline factors associated with functional decline or stability and provide insights into early correlates of functional and hormonal changes in midlife.

To examine whether smartphone-based accelerometry correlates more strongly with T levels than other functional tests in middle-aged men, data from the smartphone based accelerometry will be analyzed to compare the strength of the relationship between physical and cognitive function and T levels. The analyses will be performed separately for each test. For the STS test, associations with the number of repetitions will be compared with advanced metrics including the power (W), peak and average VV (rad/s) and movement consistency (DTW). For balance, the brief-BESTest sub-scores for SLS and foam stance (0–3) will be compared with the postural sway as measured by the COM-95% ((m^2^)^2^). For gait stability, the total duration of the TUG will be compared with the phase duration (seconds) and the angular velocity during the first turn (rad/s) measured with the smartphone. Pearson or Spearman correlation coefficients will be calculated for each factor-outcome pair. In addition, multiple linear regression models adjusted for age, BMI, physical activity level, and sleep quality will be used to compare standardized beta coefficients. These analyses will be used to determine whether smartphone-based accelerometry correlates more strongly with T levels than other functional tests in middle-aged men.

All statistical analyses will be performed using IBM SPSS Statistics (version 27) and Python (version 3.12), with a significance set at a *p*-value of <0.05. Effect sizes will be reported alongside *p*-values to provide a comprehensive understanding of the magnitude of observed associations or intervention effects.

## 3 Discussion

The main aim of this study is to explore the relationships between hormone profile, physical performance and cognitive function in healthy middle-aged men, over a one-year period. By integrating these domains within a single research framework, we aim to uncover early preclinical patterns that may signal the onset of age-related decline and enhance the understanding of how these systems interact in the early stages of aging.

Midlife is a critical yet under-researched stage in the aging process. While most studies compare younger and older adults (1), recent findings have shown that aging is a nonlinear process, with significant biologic, physical and cognitive changes occurring in the middle age (37, 38). In particular, middle-aged men are even less studied, as the menopausal transition provides a clearer biological marker for research in women. To our knowledge, no previous study has comprehensively examined hormonal, physical, and cognitive function in healthy middle-aged men.

This study offers a novel, integrative framework that aligns with the WHO healthy aging paradigm by operationalizing key areas of intrinsic capacity, defined as “the composite of all the physical and mental capacities that an individual can draw on” (39). Decline in intrinsic capacity is now recognized as a predictor of adverse outcomes such as disability, frailty, falls, hospitalization and mortality, and there is evidence that this decline can begin as early as midlife (40). However, there are few studies that have examined intrinsic capacity in this age group (40). This protocol addresses this gap by providing a comprehensive perspective on the integration between biological markers (e.g., hormone profile) with physical performance, cognitive function and mental health in middle-aged men, while also considering sleep quality to provide a broader view of the dynamics of early aging.

To address this gap, this protocol employs advanced, objective tools, including accelerometry-based sensors for physical function and the computerized NeuroTrax cognitive battery, both of which have demonstrated suitability for detecting early, subtle changes in performance among healthy adults (13, 14, 31). Recent studies highlight the value of instrumental physical assessments in this population. Baranes et al. (13) have shown that the integration of a smartphone-based accelerometery to measure sway during the Brief-BESTest reveals subtle impairments in balance between early and late middle-aged adults that are not captured by the standard assessment method. Consistent with these findings, Hayek et al. (14) demonstrated that smartphone-based STS analysis improves accuracy in detecting early mobility deterioration in middle-aged adults. While conventional timing measures of STS generally fail to detect meaningful differences between young and middle-aged adults, accelerometry-derived measures such as lower-limb power and maximal vertical velocity showed significant functional declines in the middle-aged group (14). The TUG also showed a better ability to detect differences in functional performance when applied in its instrumented form (30). In particular, parameters of the turning phase, such as turning duration and angular velocity, have been shown to be effective in detecting early balance impairments in midlife, as turning is a complex task that reflects age-related declines in postural control and has been associated with age-related decline and increased fall risk (30). The use of smartphone-based sensors, which are found in almost every pocket, could enable the development of accessible, low-cost, and scalable functional assessment tools that can be used in any clinical setting and are potentially suitable for unsupervised home monitoring without the need for an in-person physician visit. Furthermore, this protocol could help establish reference values for physical performance in healthy middle-aged adults, a gap that remains largely unaddressed in the current literature.

This one-year cohort design with three measurement points provides the opportunity to move beyond cross-sectional observations and determine whether the identified associations represent transient variability or stability. Assessing participants over time allows for the identification of enduring functional and cognitive patterns that may serve as meaningful early markers of aging and enable the development of targeted prevention strategies.

While this study protocol offers novel insights into the associations between hormones, physical performance, and cognition in midlife, several limitations should be considered. First, to minimize potential confounding factors, the study will include only healthy, community-dwelling middle-aged men and exclude those with health conditions that could affect physical or cognitive performance. Additionally, the recruitment strategy (e.g., social media, referrals), although chosen for feasibility, may introduce selection bias. Both factors should be considered when interpreting the findings, as they may limit the generalizability of the results. Second, the one-year follow-up permits evaluation of short-term stability of associations, but longer longitudinal studies are required to capture gradual hormonal and functional changes and to determine whether early markers persist or evolve over time. Third, while the focus of this protocol on androgen related-hormones (TT, FT, cBioT, SHBG) is consistent with the study aims and with previous studies in this field (15, 16, 18), future studies should consider including additional hormones (e.g., cortisol, growth hormone) to provide a more comprehensive understanding of the biological mechanisms underlying age-related functional changes in midlife.

In summary, by examining the early interactions between biological, functional, cognitive, and mental systems in midlife, using objective, tailored assessment tools that have proven effective in capturing variability in this population, our study is expected to provide new insights into the onset of aging processes. Focusing on this overlooked life stage and integrating objective assessments across multiple domains will provide the evidence base needed for early detection and support further studies to develop prevention strategies aimed at promoting healthier aging in this population.
